# Sex-specific effects of bisphenol A on the signaling pathway of ESRRG in the human placenta^†^

**DOI:** 10.1093/biolre/ioac044

**Published:** 2022-02-26

**Authors:** Zhiyong Zou, Lynda K Harris, Karen Forbes, Alexander E P Heazell

**Affiliations:** Maternal and Fetal Health Research Centre, University of Manchester, Manchester, UK; Maternal and Fetal Health Research Centre, University of Manchester, Manchester, UK; Division of Pharmacy and Optometry, Faculty of Biology, Medicine and Health, University of Manchester, Manchester, UK; Maternal and Fetal Health Research Centre, University of Manchester, Manchester, UK; Leeds Institute of Cardiovascular and Metabolic Medicine, Faculty of Medicine and Health, University of Leeds, Leeds, UK; Maternal and Fetal Health Research Centre, University of Manchester, Manchester, UK; St Mary’s Hospital, Manchester Foundation Trust, Manchester Academic Health Science Centre, Manchester, UK

**Keywords:** bisphenol A, sex specific manner, estrogen-related receptor gamma, human placenta, placental dysfunction

## Abstract

Bisphenol A (BPA) exposure during pregnancy is associated with low fetal weight, particularly in male fetuses. The expression of estrogen-related receptor gamma (*ESRRG*), a receptor for BPA in the human placenta, is reduced in fetal growth restriction. This study sought to explore whether *ESRRG* signaling mediates BPA-induced placental dysfunction and determine whether changes in the *ESRRG* signaling pathway are sex-specific. Placental villous explants from 18 normal term pregnancies were cultured with a range of BPA concentrations (1 nM–1 μM). Baseline BPA concentrations in the placental tissue used for explant culture ranged from 0.04 to 5.1 nM (average 2.3 ±1.9 nM; *n* = 6). Expression of *ESRRG* signaling pathway constituents and cell turnover were quantified. BPA (1 μM) increased *ESRRG* mRNA expression after 24 h in both sexes. ESRRG mRNA and protein expression was increased in female placentas treated with 1 μM BPA for 24 h but was decreased in male placentas treated with 1 nM or 1 μM for 48 h. Levels of 17β-hydroxysteroid dehydrogenase type 1 (*HSD17B1*) and placenta specific-1 (*PLAC1*), genes downstream of *ESRRG*, were also affected. *HSD17B1* mRNA expression was increased in female placentas by 1 μM BPA; however, 1 nM BPA reduced *HSD17B1* and *PLAC1* expression in male placentas at 48 h. BPA treatment did not affect rates of proliferation, apoptosis, or syncytiotrophoblast differentiation in cultured villous explants. This study has demonstrated that BPA affects the *ESRRG* signaling pathway in a sex-specific manner in human placentas and a possible biological mechanism to explain the differential effects of BPA exposure on male and female fetuses observed in epidemiological studies.

## Teaser

BPA affects the *ESRRG* signaling pathway in a sex-specific manner in human placentas and is a possible biological mechanism to explain the differential effects of BPA exposure on male and female fetuses observed in epidemiological studies.

## Introduction

Bisphenol A (BPA), an organic, synthetic compound, has been widely used in plastic products, including food containers, cans, beverages, baby bottles, toys, credit cards, receipts, and medical equipment since the 1950s. BPA concentrations ranging from 0.3 to 17.85 ng/mL (1–78 nM) have been observed in adult and fetal plasma, urine, breast milk, and placental tissue [1–6]. Epidemiological and animal studies have shown that maternal BPA exposure is associated with preterm birth, low birth weight, small for gestational age (SGA) infants, fetal growth restriction (FGR), and preeclampsia [3, 7–12]. These effects seem to be sex-specific, with male neonates disproportionately affected [8]. As BPA concentrations in the human placenta are four to five times higher than levels in maternal or fetal plasma [13], the placenta might play a crucial role in pregnancy disorders related to BPA exposure.

FGR is associated with a range of underlying placental conditions including maternal vascular perfusion, fetal vascular malperfusion, and placental inflammation [14]. Maternal vascular malperfusion is the most commonly observed abnormality, particularly in severe early-onset FGR [14]. Our previous studies suggest FGR is also associated with increased placental cell trophoblast apoptosis and disordered proliferation [15, 16]. However, the mechanism(s) for BPA-related, sex-specific decreases in fetal weight are still unclear and might be related to abnormal placental cell turnover within the placenta, as observed in FGR.

BPA is a weak estrogen, and although it can bind to estrogen receptor (ER)-alpha, and ER-beta, progesterone receptor, androgen receptor, and G-protein-coupled receptor 30 [17–20], BPA is a highly selective agonist for estrogen-related receptor gamma (*ESRRG*) [21–24], binding with 800 to 1000-fold higher affinity than to other receptors [17]. Furthermore, a recent study also suggests that BPA binds to *ESRRG* in a similar manner to natural *ESRRG* ligands [19]. Both mRNA and protein expression of ESRRG can be induced by short-term BPA exposure in lung cancer cell lines, breast cancer cell lines, adipocytes, hepatocytes, and zebrafish [22–26].

*ESRRG* is highly expressed in the human placenta [27, 28] and levels are reduced in FGR placentas [28–30]. Furthermore, *ESRRG* regulates a number of genes in the placenta, including cytochrome P-450 (*CYP191.1*), 17β-hydroxysteroid dehydrogenase type 1 (*HSD17B1*), 11β-hydroxysteroid dehydrogenase type 2 (*HSD11B2*), and placenta specific-1 (*PLAC1*) [29–39]. Functional studies demonstrate a role for *ESRRG* in regulating proliferation and invasion of the trophoblastic cell line, HTR8/SVneo, and stimulating the differentiation of primary cytotrophoblast from first-trimester placentas [29, 31, 40]. This evidence demonstrates that *ESRRG* can regulate many aspects of trophoblast function and suggests that dysregulated *ESRRG* signaling may contribute to the pathogenesis of FGR, as well as mediating the effects of BPA on placental dysfunction.

Sex-specific effects of BPA exposure are observed in the offspring’s sex differentiation, abnormal neurobehavioral outcomes, skeletal muscle hypertrophy, and cardiovascular function in animal models, and the genes mediating these effects include *Esrrg* and *Cyp191.1* [41–54]. Even though some previous studies have explored the effects of BPA exposure on the human placenta by using trophoblast cell lines or animal models [55–72], only one recent study has investigated the sex-specific effects of BPA on the placenta: Mao et al. used a mouse model of BPA exposure in pregnancy and reported no sex-specific effects [73]. Therefore, the potential sex-specific effects of BPA on the human placenta are yet to be investigated. In addition, only one study has explored the effects of BPA exposure on *ESRRG* expression, using the trophoblast choriocarcinoma cell line JEG-3 (RRID:CVCL_0363), which was derived from the placenta of a male fetus [72]. They observed that 10 nM BPA reduced DNA synthesis after 24 and 48 h of exposure, which was mediated in part by *ESRRG* signaling. As JEG-3 cells are neoplastic, they are not the most physiologically relevant model for study, thus further work is required to establish the effects of BPA on the *ESRRG* pathway and trophoblast cell turnover in the human placenta.

As *ESRRG* has been implicated as a regulator of signaling pathways underlying placental dysfunction in FGR placentas [29, 31] and BPA signals through *ESRRG*, we hypothesized that BPA exposure might initiate or exacerbate placental dysfunction via activation of *ESSRG*. Therefore, this study will explore whether a range of doses of BPA (1 pM, 1 nM, and 1 μM) can alter *ESRRG* signaling and cell turnover in the human placental explants and assess whether the responses are sex-specific.

## Methods

Unless specified, reagents were purchased from Sigma (Sigma, UK).

### Placental collection

This study was approved by the Research Ethics Committee (08/H1010/55 + 5) and informed consent was collected from all participants. All placental samples were collected from Saint Mary’s Hospital, Manchester, and were obtained within 30 min of delivery. In our study, appropriate for gestational age (AGA) (*n* = 18) was defined as an individualized birth weight ratio between the 10th and 90th centile calculated by gestation-related optimal weight software (Gestation Network; Birmingham, UK, www.gestation.net) [74]. All participants were non-smokers, less than 40 years of age and had a body mass index (BMI) of <30 kg/m^2^. Women with pregnancy complications such as preeclampsia, chronic hypertension, renal disease, collagen vascular disease, premature rupture of membranes, and pregnancies complicated with fetal anomalies or chromosomal abnormalities were excluded from this study.

### Placental explant culture

Placental explants were prepared as previously described [16]. Briefly, six pieces of villous tissue (2 cm^3^ each) were sampled from normal term placentas from 18 AGA infants (nine male and nine female), which were further excised as 2 mm^3^ placental explants and cultured in Netwells (Corning Inc., NY, USA) with 1.5 ml culture medium, composed of 1:1 of Dulbecco Modified Eagle Medium (DMEM; Gibco, UK)/Ham’s F12 (Gibco, UK) supplemented with penicillin (0.6 mg/l), streptomycin (100 μg/ml), L-glutamine (0.292 g/l), and 10% fetal calf serum (Gibco, UK).

The environment-related BPA levels (1 nM) and a higher (1 μM), and a lower BPA concentration (1 pM) were selected for use in this study to explore the effects of a range of relevant BPA concentrations. BPA was dissolved in 50% (v/v) ethanol (with 50% sterile PBS) at a stock concentration of 1 mM and stored at 4 °C. The stock solution was further diluted into different concentrations of BPA with culture medium prior to addition to the villous explants. Fresh BPA dilutions in culture medium were prepared for every experiment, and all products used during the culture, such as 12 well plates, netwells, and tips, were BPA-free.

Explants were cultured at 21% O_2_ (5% CO_2_, 95% air at 37 degrees) for 24 or 48 h. The villous explants treated with 0.05% (v/v) ethanol were considered as the control group. Conditioned-culture medium and villous tissue were collected at 24 or 48 h of culture and were processed for enzyme-linked immunoassay (ELISA), ribonucleic acid (RNA) or protein extraction, or immunohistochemistry (IHC) analysis.

### RNA extraction and reverse transcription polymerase chain reaction (RT-PCR)

Tissue was homogenized (using a handhold homogenizer (SHM1, UK)) and total RNA was extracted using a miRNeasy mini kit (QIAGEN, Germany), following the manufacturer’s instructions. 500 ng RNA was converted to cDNA using an AffinityScript Multiple Temperature cDNA according to the manufacturer’s instructions. The PCR primer sequences (Eurofins, UK) for *ESRRG* and *RPLP0* (60S acidic ribosomal protein P0) are listed in Supplementary Table S1. *RPLP0* was used as a housekeeping gene and an endogenous control, as it was stably expressed in placental tissues [29]. Powerup SYBR Green Master Mix (Thermo Fisher Scientific, USA) was used in the PCR reaction and an Applied Biosystems Step-one System (Thermo Fisher Scientific, USA) was used to run the reaction with an annealing temperature of 60 °C, followed by a melt curve step. The fold expression was calculated by the 2^−△△CT^ method.

### Analysis of human chorionic gonadotropin secretion and lactate dehydrogenase

Human chorionic gonadotropin (hCG) is expressed in the placental trophoblast and is a marker of cytotrophoblast differentiation. Structurally intact hCG (alpha and beta unit) was quantified in the explant-conditioned culture medium and in villous explants, which were collected at 24 or 48 h and lysed in 0.3 M NaOH. The hCG level in the conditioned-cultured medium was measured using an hCG ELISA kit (DRG Diagnostics, Marburg, Germany), and a BioRad protein assay (Bio-Rad Laboratories, Hempstead, UK) was used to detect the protein content in the villous explants according to the manufacturer’s instructions. The hCG secretion was expressed as mIU/ml/mg explant protein/h.

Lactate dehydrogenase (LDH) is an enzyme that converts lactate to pyruvate in live cells; LDH is released from necrotic cells, thus is a marker of cellular viability [75]. LDH release in the explant-conditioned culture medium was quantified as a proxy measure of necrosis in the explants, using a cytotoxicity detection kit (Roche Diagnostics, Mannheim, Germany), according to the manufacturer’s instructions. LDH release was expressed as absorbance units/mg explant protein/h.

### BPA content enzyme-linked immunosorbent assay

Six fresh placentas from three male and three female infants were collected and stored at −80 °C prior to sample preparation. Briefly, placental tissue (approximately 1 g) was homogenized in 4 ml dH_2_O at room temperature, and 8 μl acetic acid was added to acidify the homogenate. After supplying 4 ml ethyl acetate, the homogenate was vortexed and centrifuged at 12 000 g for 3 min. The organic phase was collected and dried with nitrogen gas, and the dried residue was dissolved in 20 μl ethanol (96%, v/v) with the addition of a 500 μl sample dilution buffer. Samples were centrifuged at 10 000 g for 5 min, and then the supernatant was collected for analysis by ELISA (Estrogen BPA Environmental ELISA Kit, ab175820, Abcam, UK). The ELISA has a sensitivity for BPA measurement ranging from 0.003 to 1000 ng/ml.

Around 100 μl sample or standard were added to a 96 well ELISA plate in duplicate, and 100 μl horseradish peroxidase (HRP)-conjugate solution was added to the wells for 2 h at room temperature. After washing the wells three times with washing buffer, 200 μl TMB (3,3′,5,5′-Tetramethylbenzidine) substrate was added for 30 min at room temperature. Around 50 μl 2 N sulfuric acid was added into the wells to stop the reaction, and the absorbance was immediately read at 450 nm. The accompanying standard curve was used to calculate the sample BPA concentrations.

### Immunostaining

Placental explants were fixed in 4% neutral buffered formalin overnight at 4°C and embedded in paraffin wax. A total of 5 μm sections were cut and transferred onto the slides pre-coated with poly-l-lysine. After being deparaffinized, the slides were treated for antigen retrieval by microwave boiling (800 W, 10 min) and then incubated with 3% (v/v) hydrogen peroxide for 10 min. Slides were incubated with the non-immune block (10% goat serum and 2% human serum in 0.1% TBST (TBS-Tween-20) for 30 min at room temperature and then incubated with a polyclonal antibody against ESRRG (Abcam 215947, 10 μg/ml), a monoclonal antibody against Ki67 (Dako, 0.17 μg/ml), or M30 (Roche, 0.13 μg/ml) overnight at 4 °C. The negative control was an isotype-specific non-immune rabbit or mouse IgG used at the same concentration as the primary antibodies. Immunostaining for Ki67 and M30 was carried out as markers of cycling cells and hence were proxy markers of proliferation and apoptosis, respectively [16, 76]. The secondary antibody (biotin-conjugated goat anti-mouse or anti-rabbit antibodies, Dako-Cytomation, UK, 3.85 μg/ml) was applied and incubated for 30 min at room temperature followed by incubation with avidin-peroxidase (5 μg/ml) for 30 min. Chromogenic substrate diaminobenzidine (DAB; Sigma-Aldrich, UK) was applied to the sections for between 2 and 10 min; color development was monitored under the microscope. All slides were counterstained with Harris’s hematoxylin (Sigma-Aldrich, UK) for 5 min and then differentiated with acid alcohol for 2 s. All villous explants for comparison were stained in the same batch, and all negative control samples had no immunoactivity.

### Statistical analysis

All data are presented as the mean ± standard deviation (SD) (normally distributed) or median ± interquartile range (IQR) (non-normally distributed); a Shapiro–Wilk normality test was used to determine whether the data were normally distributed. Statistical analysis was undertaken with GraphPad Prism version 7.0 (GraphPad Software, USA). Data were assessed using one sample Wilcoxon test or Kruskal–Wallis, followed by a Friedman multiple comparison test for non-parametric data. QuPath version 0.2.3 (developed by the University of Edinburgh) was used to analyze the IHC staining results [77]. The detection of positively stained cells was used to quantify the extent of Ki67 and M30 staining, which was expressed as a percentage of the total number of cells. For ESRRG protein staining, the percentage of DAB-positive area to total tissue area was calculated. A *P* value < 0.05 was considered statistically significant.

## Results

### Demographic characteristics

The demographic characteristics of participants are listed in Table 1. The age, BMI, gestational age, and ethnicity of pregnant women were comparable between the male and female fetuses group, as well as the fetal weight and Apgar score.

**Table 1:** Demographics of pregnant women and fetuses

Characteristics	Male group (*n* = 9)	Female group (*n* = 9)	*P* value
Birth weight (g)	3410.0 ± 625.0	3436.0 ± 391.0	NS
Gestational age at delivery (weeks)	39.2	38.9	NS
Maternal age (years)	32.8 ± 2.8	29.9 ± 4.8	NS
Parity (primary/multiple)	2/7	1/8	
BMI (kg/m^2^)	26.4 ± 3.3	24.7 ± 5.0	NS
Ethnicity			
White British	6 (66.7%)	4(44.4%)	NS
Indian	0	1(11.1%)	
Pakistani	1(11.1%)	2(22.2%)	
Black	1(11.1%)	1(11.1%)	
Other	1(11.1%)	1(11.1%)	
Apgar score (at 5 min)	10	10	NS

Data were presented as mean ± SD or median ± IQR. NS, no significance.

### BPA alters ESRRG expression in a sex-specific manner in term villous explants

**Figure 1:**
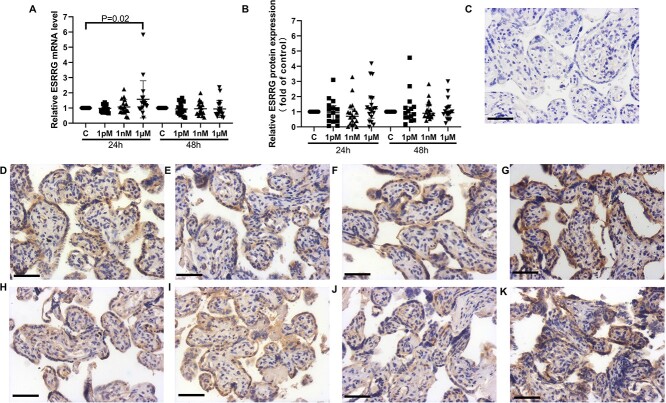
Effects of BPA exposure on expression of *ESRRG* in term villous explants. (A) mRNA expression of ESRRG. (B) Quantification of ESRRG immunostaining. (C–J) Representative images of immunostained cultured villous explants. (C) Negative control. (D–G) 24 h of culture; (H–K) 48 h of culture. (D, H) Controls (0.05% (v/v) ethanol); (E, I) 1 pM BPA; (F, J) 1 nM BPA; (G, K) 1 μM BPA. One sample Wilcoxon test, median ± IQR. *n* = 18. Bar = 50 μm.

Baseline BPA concentrations in the placental tissue used for explant culture ranged from 0.04 to 5.1 nM (average 2.3 ±1.9 nM; *n* = 6). The BPA concentrations were similar in placentas from male and female infants (male mean 2.2 ±2.0 nM vs. 2.4 ± 1.8 nM in females, Supplementary Figure S1). The effects of in vitro BPA treatment (1 pM–1 μM above baseline), on the mRNA and protein expression of *ESRRG* in term placental villous explants cultured for 24 or 48 h was then examined (Figure 1). mRNA expression of  *ESRRG* was significantly increased following exposure to 1 μM BPA for 24 h, but there was no change in expression after treatment with 1 pM or 1 nM BPA (Figure 1A). After 48 h, BPA treatment did not significantly alter mRNA or protein expression of ESRRG at any concentration (Figure 1A and B). There was no change in the localization of ESRRG following BPA treatment (Figure 1D–K), which was predominantly found in the syncytiotrophoblast layer and endothelium of the villous.

**Figure 2:**
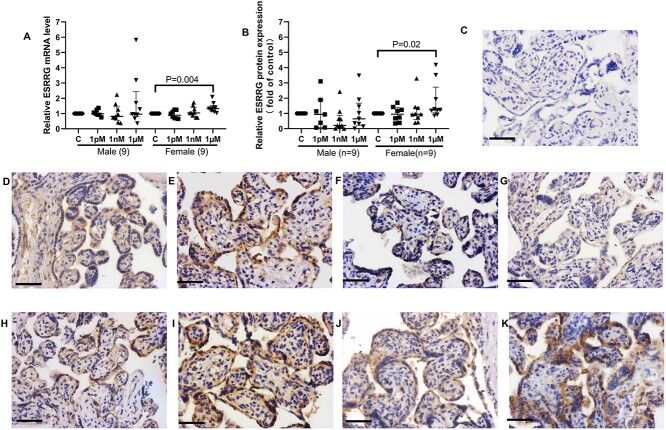
mRNA and protein expression of ESRRG in term villous explants from male and female fetuses after BPA exposure for 24 h. (A) mRNA expression of *ESRRG*. (B) Quantification of ESRRG immunostaining. (C) Negative control. Representative images of immunostained villous explants from male infants (D–G) and female fetuses (H–K). (D, H) Controls (0.05% (v/v) ethanol). (E, I) 1 pM BPA. (F, J) 1 nM BPA. (G K) 1 μM BPA. One sample Wilcoxon test, median ± IQR. Bar = 50 μm.

When data from male and female placentas were separated by sex, BPA treatment led to a sex-specific alteration of *ESRRG* expression; 1 μM BPA significantly increased the mRNA level of *ESRRG* in female placentas after 24 h (Figure 2A, *P* < 0.01), which was consistent with the protein expression data (Figure 2B, *P* < 0.05). We also observed that ESRRG protein staining is mainly localized in the cytoplasm of syncytiotrophoblast, and while overall levels increased, localization was unaffected by BPA treatment (Figure 2D–K). In the untreated placental explants, the mRNA levels of *ESRRG* and its downstream genes were comparable between male and female infants (Supplementary Figure S2).

**Figure 3:**
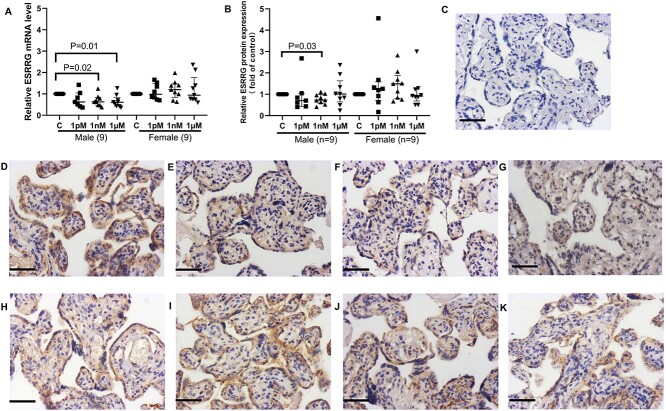
mRNA and protein expression of ESRRG in male or female villous explants treated with BPA for 48 h. (A) mRNA expression of *ESRRG*. (B) Quantification of ESRRG immunostaining. (C) Negative control. Representative images of immunostained placental explants from male fetuses (D–G) and female infants (H–K). (D H) Control (0.05% (v/v) ethanol). (E, I) 1 pM BPA. (F, J) 1 nM BPA. (G, K) 1 μM BPA. One sample Wilcoxon test, median ± IQR. Bar = 50 μm.

After 48 h, the mRNA level of *ESRRG* was significantly decreased in placental explants from male infants following treatment with 1 nM or 1 μM BPA (Figure 3A). Quantification of ESRRG protein expression confirmed the reduction of ESRRG with 1 nM BPA treatment in male villous explants (Figure 3A and B, *P* < 0.05). The localization of ESRRG protein in male placentas (Figure 3D–G) did not appear to differ from female placentas (Figure 3H–K).

### BPA alters mRNA expression of genes downstream of *ESRRG* in a sex-specific manner

**Figure 4:**
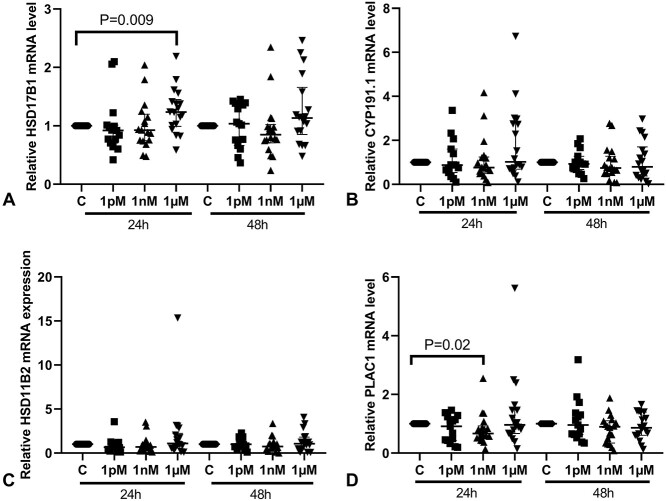
Effects of BPA on the mRNA levels of downstream genes of *ESRRG*. (A) *HSD17B1*; (B) *CYP191.1*; (C) *HSD11B2*; (D) *PLAC1*. One sample Wilcoxon test; *n* = 18. Median ± IQR. *CYP191.1*, cytochrome P-450; *HSD17B1*, 17β-hydroxysteroid dehydrogenase type 1; *HSD11B2*, 11β-hydroxysteroid dehydrogenase type 2; *PLAC1*, placenta specific-1.

To assess the effects of BPA on *ESRRG* signaling, we measured the mRNA expression of several genes downstream of *ESRRG*, including *HSD17B1*, *CYP191.1*, *HSD11B2*, and *PLAC1*. In the villous explants from both male and female placentas, the mRNA level of *HSD17B1* was significantly increased after treatment with 1 μM BPA for 24 h (Figure 4A, *P* < 0.01). mRNA expression of *CYP191.1* and *HSD11B2* was unchanged at 24 h following BPA treatment (Figure 4B and C). The mRNA level of *PLAC1* was significantly reduced after treatment with 1 nM BPA for 24 h, compared to control explants (*P* < 0.05, Figure 4D). No changes in mRNA expression of these four genes were observed after 48 h of BPA treatment.

**Figure 5:**
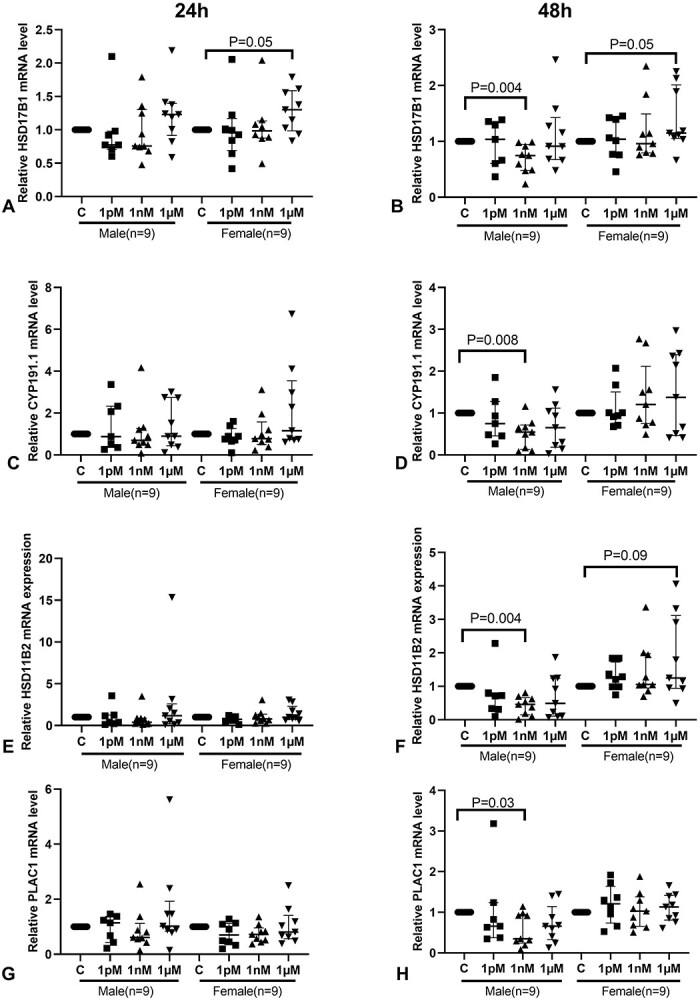
The sex-specific effects of BPA on the mRNA levels of downstream genes of *ESRRG*’. (A) (*HSD17B1*, 24 h) and (B) (*HSD17B1*, 48 h); (C) (*CYP191.1*, 24 h) and (D) (*CYP191.1*, 48 h); (E) (*HSD11B2*, 24 h) and (F) (*HSD11B2*, 48 h). (G) (*PLAC1*, 24 h) and (H) (*PLAC1*, 48 h), mRNA levels. One sample Wilcoxon test, median ± IQR. *CYP191.1*, cytochrome P-450; *HSD17B1*, 17β-hydroxysteroid dehydrogenase type 1; *HSD11B2*, 11β-hydroxysteroid dehydrogenase type 2; *PLAC1*, placenta specific-1.

Sex-specific effects of BPA on the mRNA levels of these four genes were also observed. In placentas from female fetuses, compared to the control group, the mRNA expression of *HSD17B1* increased following treatment with 1 μM BPA for 24 h (*P* = 0.05, Figure 5A) or 48 h of culture (*P* = 0.05, Figure 5B). Meanwhile, the mRNA expression of four downstream genes was significantly decreased in the villous explants from male fetuses following treatment with 1 nM BPA for 48 h (Figure 5B, D, F, and H).

### BPA effects on trophoblast differentiation and necrosis

**Figure 6:**
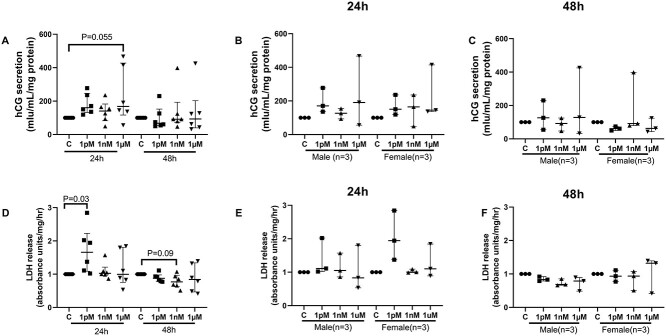
hCG and LDH levels in explant culture medium following BPA exposure. (A) hCG and (D) LDH levels in the culture medium from all villous explants after BPA treatment for 24 or 48 h (*n* = 6). (B) hCG levels in culture medium from explants from male and female for 24 h of culture, and (C) for 48 h of culture. (E) LDH levels in culture medium from explants from male and female for 24 h of culture, and (F) for 48 h of culture. *n* = 6. Median ± IQR, one sample Wilcoxon test.

BPA treatment did not change the level of hCG secreted into the culture medium at any concentration tested at 24 or 48 h (Figure 6A). LDH release was modestly but significantly increased after treatment with 1 pM BPA for 24 h, but levels were comparable in the villous explants cultured for 48 h (Figure 6D, *P* < 0.05). There was no evidence of sexually dimorphic responses in the hCG secretion or LDH release in response to BPA exposure (Figure 6B, C, E, and F).

### BPA effects on Ki67 and M30 expression

**Figure 7:**
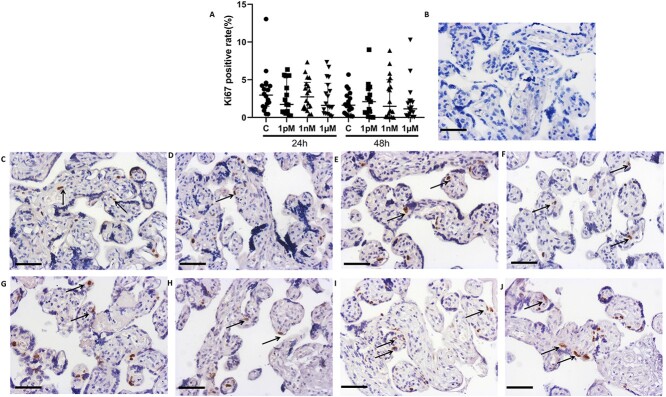
Effects of BPA on the percentage of cells in cycle in villous explants cultured for 24 or 48 h. (A) Quantification of Ki67 staining. (B) Negative control. Representative images of ki67 staining in the placental explants cultured for 24 h (C–F) or 48 h (G–J). (C, G) Control group (0.05% ethanol); (D, H) 1 pM BPA; (E, I) 1 nM BPA; (F, J) 1 μM BPA. Black arrow, Ki67 positive cells. Kruskal-Wallis test, median ± IQR. *n* = 18. Bar = 50 μm.

**Figure 8:**
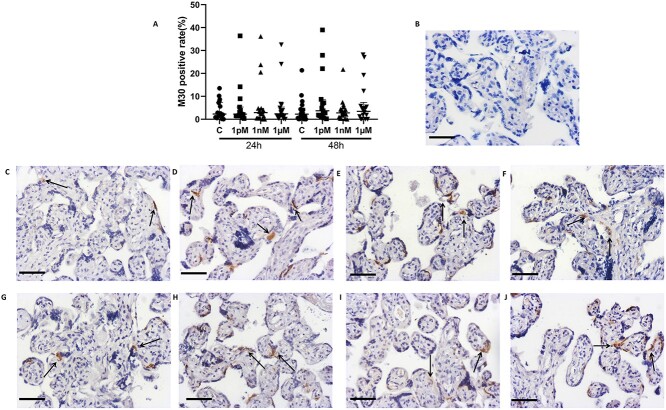
Effects of BPA on the percentage of apoptotic cells in villous explants cultured for 24 or 48 h. (A) Quantification of M30 staining. (B) Negative control. Representative images of M30 staining after 24 (C–F) or 48 h (G–J) of BPA exposure. (C, G) Control (0.05% (v/v) ethanol); (D, H) 1 pM BPA; (E, I) 1 nM BPA; (F, J) 1 μM BPA. Black arrow, M30 positive cells. Kruskal-Wallis test, median ± IQR. *n* = 18. Bar = 50 μm.

Compared to the control group, the percentage of cells in cycle (Ki67-positive cells) and apoptotic cells (M30-positive cells) were not affected by any concentration of BPA at 24 and 48 h in the cultured explants (Figures 7 and 8), and no sexually dimorphic responses were observed in these explants with BPA exposure (Supplementary Figures S3–S6).

## Discussion

In this study, we have explored, for the first time, the sex-specific effects of BPA on *ESRRG* signaling in the human term placenta. This finding provides some biological evidence to aid understanding of the differential effects of BPA exposure on male and female fetuses observed in epidemiological studies.

### The sex-specific effects of BPA in the human placenta

The mean BPA concentration in the fresh placental tissue was consistent with reported BPA concentrations in human placental tissues [5, 6, 78, 79]; this confirms the exogenous BPA concentrations applied in this study were physiologically relevant. We observed that short-term treatment with 1 μM BPA altered *ESRRG* expression. In HeLa cells, BPA has been shown to bind to *ESRRG*, maintaining its high constitutive signaling activity without further increasing its expression [17]. However, our results are similar to previous studies in breast and lung cancer cell lines, which reported that both ESRRG mRNA and protein expression could be increased by short-term BPA exposure [22, 24–26]. The only previous study in the trophoblast cells (JEG-3 cells) to examine the relationship between *ESRRG* and BPA showed that siRNA-mediated suppression of *ESRRG* partially prevented the reduction [3H]-thymidine DNA incorporation induced by BPA treatment [72]. We have confirmed the ability of BPA to signal through *ESRRG* using a more physiological culture model but also report sex-specific effects of BPA exposure.

The findings that 1 μM BPA significantly increased ESRRG mRNA and protein expression in the female placentas, but the reduced expression in male placentas suggests that low-dose BPA exposure can alter gene expression in the human placentas in a sex-specific manner. The concept that BPA exposure can alter gene expression is supported by data from Mao et al., who used RNA-seq to assess mouse placentas exposed to BPA before or during pregnancy. In these experiments BPA altered expression of 13 genes (*Actn2*, *Calm4*, *Coch*, *Cxcl14*, *Ear2/NR2F6*, *Efcab2*, *Epdr1*, *Gdf10*, *Gm9513/PATE1*, *Guca2a*, *MMP3*, *Rimklb*, *and Sfrp4*). However, these analyses did not find any sex differences [73]. Numerous previous studies have used trophoblastic cell lines, including JEG-3, BeWo cells, 3A, and HTR8-SVneo cells, or primary cytotrophoblast cells, to explore the effects of BPA exposure on trophoblast functions and intracellular signaling pathways [59, 60, 62, 67, 68, 70, 72, 80, 81]. However, as these cell lines are derived from a single placenta, and the sex of that placenta is not always specified, these studies are unable to interrogate the sex-specific effects of BPA on trophoblast function and cell signaling.

We also report that the expression of several genes downstream of *ESRRG* is altered by BPA exposure in a sex-specific manner and responded consistently with changes in ESRRG mRNA and protein expression. Previous studies have examined the induction of *HSD11B2* mRNA expression in trophoblastic cell lines or primary cytotrophoblast following BPA exposure without exploring the sex influences [60, 82]. Interestingly, prenatal BPA exposure downregulated the mRNA levels of *Cyp19a1* in the brain of male offspring, whereas *Cyp19a1* mRNA was increased in the female brain, with increased anxiety-like behavior and decreased exploratory behavior observed in both male and female F1 rats [45]. The effects of BPA on *CYP191.1* mRNA expression have been reported in JEG-3 cells, a cell line derived from the placenta of a male fetus. One study showed that mRNA expression of *CYP191.1* was significantly decreased in JEG-3 cells after exposure to 5 μM, 10 μM, or higher BPA concentrations for 24 h [68]; however, another study using JEG-3 cells found that both the mRNA and protein expression of CYP1A1 was significantly increased after treatment with 1, 10, and 50 μM BPA, but CYP191.1 mRNA and protein expression were significantly downregulated following treatment with 1, 10, or 50 μM BPA at 72 h [67]. Compared to these previous studies, we also observed reduced mRNA expression of *CYP191.1* in male placental explants at the lower BPA concentration of 1 nM. Our data suggest that the sex-specific effects of BPA on ESRRG translate to reductions in *CYP191.1* and *HSD11B2* mRNA expression in the placentas from male infants.

To our knowledge, there are no studies that explore the expression of *HSD17B1* and *PLAC1* in the placenta following BPA exposure, and our results confirmed that these two genes could be regulated by BPA in human placentas in a sex-specific manner. Furthermore, as the sex-specific effects of BPA on *ESRRG* signaling pathways were mainly observed following treatment with BPA at levels that mimic environmental exposure (1 nM), this suggests that more attention should be paid to male fetuses and their placentas following maternal BPA exposure during the pregnancy. It is also important to note that BPA could target these downstream genes directly, not through *ESRRG*, and exert effects on other genes; further work is needed to explore these effects to determine whether they are a direct or indirect result of BPA treatment.

### Why might there be a difference in male and female placentas?

It is currently unclear how BPA regulates *ESRRG*-related genes in a sex-specific manner; one potential mechanism is through epigenetics, such as the regulation of DNA methylation. Although the epigenetic modifications of genes in our samples are unknown, studies support the possibility that BPA treatment can lead to epigenetic modifications in the liver and brain of mice [45]. Maternal BPA exposure reduced the mRNA expression of *Dnmt3b* and *Kmt2c* in the livers of male offspring on a postnatal day 1, but significantly increased the expression of *Kmt2c* in the livers of female offspring due to epigenetic modifications [83]. Another study also found that BPA could modulate the expression of genes within the ER signaling pathway, including *Esrrg*, in a sex-specific manner in the brains of mice; this was mediated by epigenetic modifications [41].

BPA exposure has also been shown to alter DNA methylation status in the placentas of humans and mice, and in the first-trimester trophoblast cell line, HTR8/SVneo, a sex-unspecified cell line; affected genes included those which regulate proliferation and migration, such as *LINE-1*, *HLA-DRB6*, *HDAC4*, *HLA-DRB1*, and *WNT-2* [82, 84–87], but only a few studies have explored sex-specific differences in placental gene methylation [84, 88]. Song et al. showed there was no sex association between BPA exposure and the hypermethylation of *HLA-DRB6* in the human placenta but only examined six placentas with (*n* = 3) or without (*n* = 3) detectable BPA concentrations [84]. However, Vilahur et al. studied 192 placentas and found that the total effective xenoestrogen burden (TEXB) biomarker, TEXB-alpha, correlated with decreased AluYb8 DNA methylation in male placentas, but not in female placentas [88], providing more evidence that maternal BPA exposure may alter DNA methylation in human placentas in a sex-specific manner.

BPA (1 nM) has also been reported to decrease the CpG methylation of several gene promoters associated with metabolic and oxidative stress in the HTR8/SVneo cell line, including *GSR*, *DNAJA1*, *PRDX2*, *GPX3*, *DDIT3*, *HERPUD1*, *INSIG1,* and *MBTPS1* [82]. Given that *ESRRG* plays an important role in regulating placental metabolism, mitochondrial function, and oxidative stress in the cytotrophoblast [28], altered methylation of promotors within the *ESRRG* gene might be one of the mechanisms by which BPA exerts its sex-specific effects (Supplementary Figure S7).

Another possible explanation may be due to the difference in BPA metabolism between male and female placentas. BPA is metabolized by UDP-glucuronosyltransferase (UGT), which is expressed in the human kidney, liver, and placenta; all *UGT2B* isoforms and *UGT1A* are expressed in the human placenta [89, 90]. Several studies have reported that blood BPA concentration is higher in males than the females, both in mice and humans [91], which correlates with the observation that *Ugtb1* is more highly expressed in the livers of female mice compared to males [91, 92]. Furthermore, the level of circulating BPA metabolites is higher in women than men [93], suggesting that elevated UGT1A expression leads to more rapid BPA metabolism in females. Sex-specific gene expression of UGT has been reported in the kidney and liver of mice but was not observed in the placenta [94]. To our knowledge, sex differences in UGT expression in the human placenta have not been well studied. Further studies exploring the placental UGT expression might be helpful to explain the sex-specific effects observed in our study.

### How does this help our understanding of the pathogenesis of FGR in human pregnancy?

Our ultimate aim is to understand the mechanisms underlying placental dysfunction observed in FGR, with a particular focus on the role of *ESRRG* [95]. The sex-specific effects of BPA on *ESRRG* signaling we observed might begin to provide a biological explanation for the observed relationship between low fetal weight in male neonates and maternal BPA exposure seen in epidemiological studies. Even though the relationship between fetal weight and maternal BPA exposure has been considered controversial by some [7, 8, 96], there are other studies that show a significant relationship between low fetal weight and BPA exposure in both mice and humans [7–9]. In mice, prenatal BPA exposure can cause FGR, but sex differences were not explored [9]. In human studies, maternal BPA exposure increased the risk of reduced fetal weight and SGA in male infants [7, 8]. As *ESRRG* is significantly decreased in human placentas from FGR pregnancies, and reduced expression of *ESRRG* is associated with abnormal proliferation and invasion of first-trimester trophoblastic cell lines [29, 31], compounds which decrease *ESRRG* expression, such as BPA, may act through *ESRRG* to induce placental dysfunction and FGR. Furthermore, reduced fetal weight in male neonates following maternal BPA exposure might be a consequence of reduced placental expression and impaired signaling through *ESRRG* (Supplementary Figure S7). Our results suggest that male placentas may be more vulnerable to maternal BPA exposure than female placentas. Although no changes in the percentage of Ki67 positive cells and M30 positive cells were observed following short term BPA exposure in this study, previous studies have described anti-proliferative and apoptotic effects of BPA on term primary cytotrophoblast or BeWo cells by measuring [3H]-thymidine incorporation or quantifying changes in apoptotic markers, including M30 and tumor-necrosis factor-alpha protein [61, 72]. In this study, we quantified Ki67 protein expression using immunostaining, which is a proliferative marker that is expressed in all active phases of the cell cycle (G1, S, G2, and M); we also used an anti-M30 antibody, which is directed against a specific epitope of cytokeratin 18 that is formed by early caspase cleavage in apoptotic cells [97, 98]. Additional markers of apoptosis and proliferation should be explored to further investigate the effects of BPA on trophoblast turnover in cultured explants.

The increase in hCG secretion following 1 μM BPA treatment is consistent with previous studies, which found BPA can induce the secretion and mRNA expression of hCG in terms of primary trophoblast and BeWo cells [59, 60, 99], and first-trimester villous explants [58]. More studies are needed to confirm the relationship between trophoblast differentiation, hCG secretion, and BPA-induced *ESRRG* signaling. In contrast, BPA did not alter LDH release from explants at 1 nM or 1 μM, after 24 or 48 h of culture; however, BPA increased LDH release from explants treated with 1 pM BPA for 24 h. Prior studies have indicated that BPA exposures between the ranges of 100 pM–50 μM did not alter LDH levels in the culture medium of JEG-3 cells [67, 72]. Interestingly, the JEG-3 cell line is derived from a male placenta, and the high LDH release we observed mainly occurred in BPA-exposed female placentas. These data might suggest that female placentas may be more sensitive to low-dose BPA exposure than male placentas.

### Strengths and limitations of this study

The findings of this study are strengthened by the use of a relevant range of BPA concentrations, which included those reflecting environmental exposure, in a controlled experimental protocol using BPA-free reagents and plasticware. The use of fresh term placental villous explants, as opposed to transformed cell lines or those derived from neoplasias, represents the most physiologically appropriate ex vivo culture model available and allows comparison to be made with other studies which used this approach. Nevertheless, there are several limitations in this study, including the relatively small sample size. In this study, there was some variability in hCG secretion, LDH release, and the percentage of Ki-67 or M30 positive cells, reflecting inherent differences in the experimental tissues. However, all reported values are within the ranges reported by our previous studies or the data from others [76, 100–102]. Nevertheless, our study recruited 18 placentas for villous explant culture; a further study with a larger sample size may be needed to identify potential BPA effects on trophoblast cell turnover using additional proliferative and apoptotic markers, particularly if the influence of maternal plasma BPA levels during pregnancy are also to be taken into account. It would also be worthwhile to further explore the non-monotonic response to BPA in the explant model by testing a wider range of physiologically relevant BPA concentrations. Furthermore, the influence of BPA exposure on other aspects of placental function, e.g., hormone secretion and nutrient transport, and BPA effects on trophoblast turnover in early pregnancy, will also be important areas for study. Finally, the effects of chronic rather than acute BPA exposure may more accurately represent environmental conditions to which the placenta is exposed in utero.

## Conclusion

In summary, we demonstrated that BPA exposure altered *ESRRG* signaling pathways in a sex-specific manner in human placental explants, but did not affect the basal level of hCG or LDH secretion, nor the number of placental cells in cycle or undergoing apoptosis. As BPA exposure alters *ESRRG* expression, and *ESRRG* regulates many aspects of trophoblast function, BPA may act through *ESRRG* to induce or enhance placental dysfunction and contribute to the pathophysiology of FGR.

## Supplementary Material

ZOU_Sup_1_ioac044

ZOU_Sup_2_ioac044

ZOU_Sup_3_ioac044

ZOU_Sup_4_ioac044

ZOU_Sup_5_ioac044

ZOU_Sup_6_ioac044

ZOU_Sup_7_ioac044

supplementary_table_1_ioac044

## Data Availability

The data underlying this article will be shared at reasonable request to the corresponding author.
