# Supplementary figures and images for: Sex-specific effects of bisphenol A on the signaling pathway of ESRRG in the human placenta
Source: Biol Reprod. 2022 Feb 26;106(6):1278–91. doi: 10.1093/biolre/ioac044 (PMC9198953; doi:10.1093/biolre/ioac044)

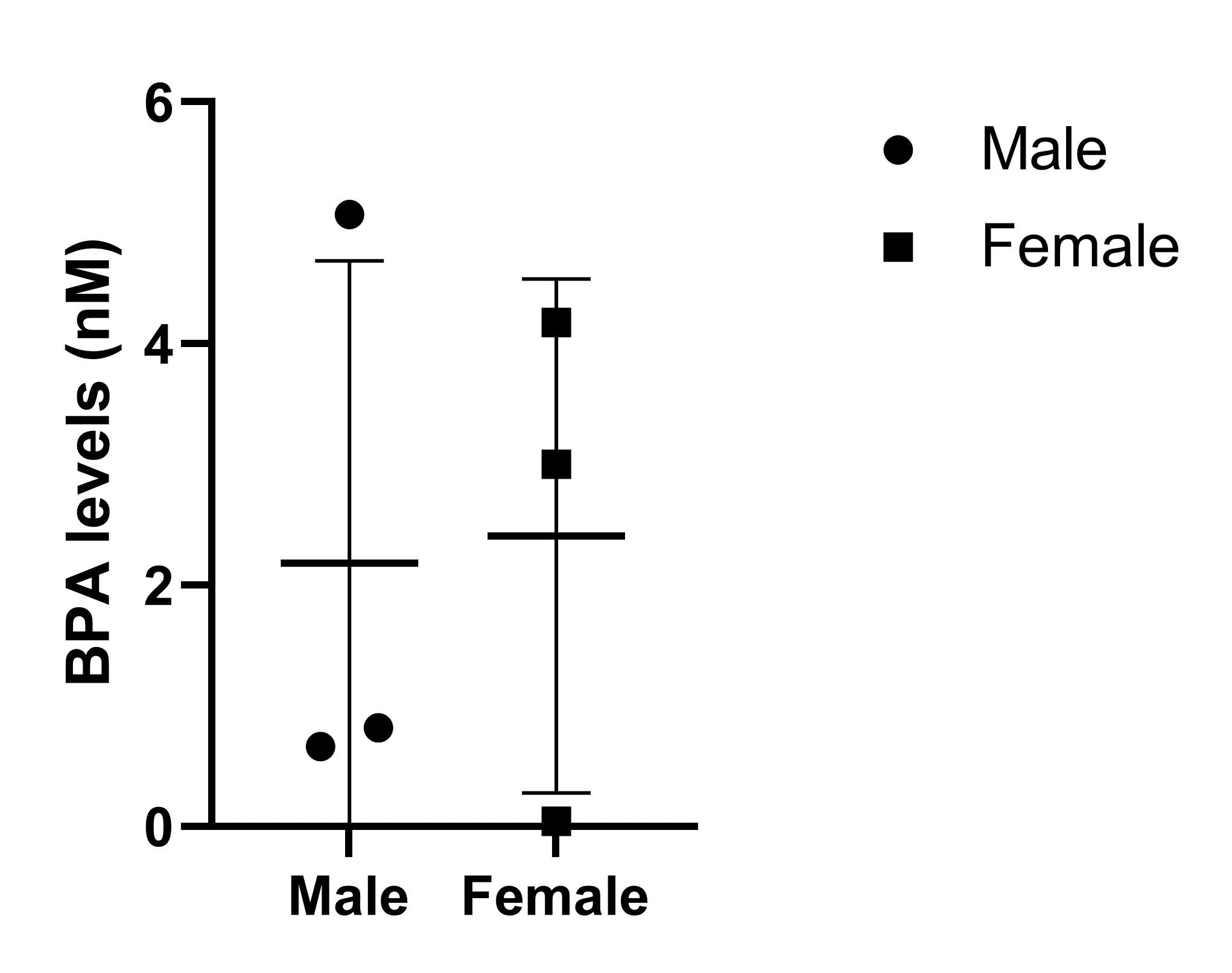

Supplement: ZOU_Sup_1_ioac044 [file zou_sup_1_ioac044.jpeg]

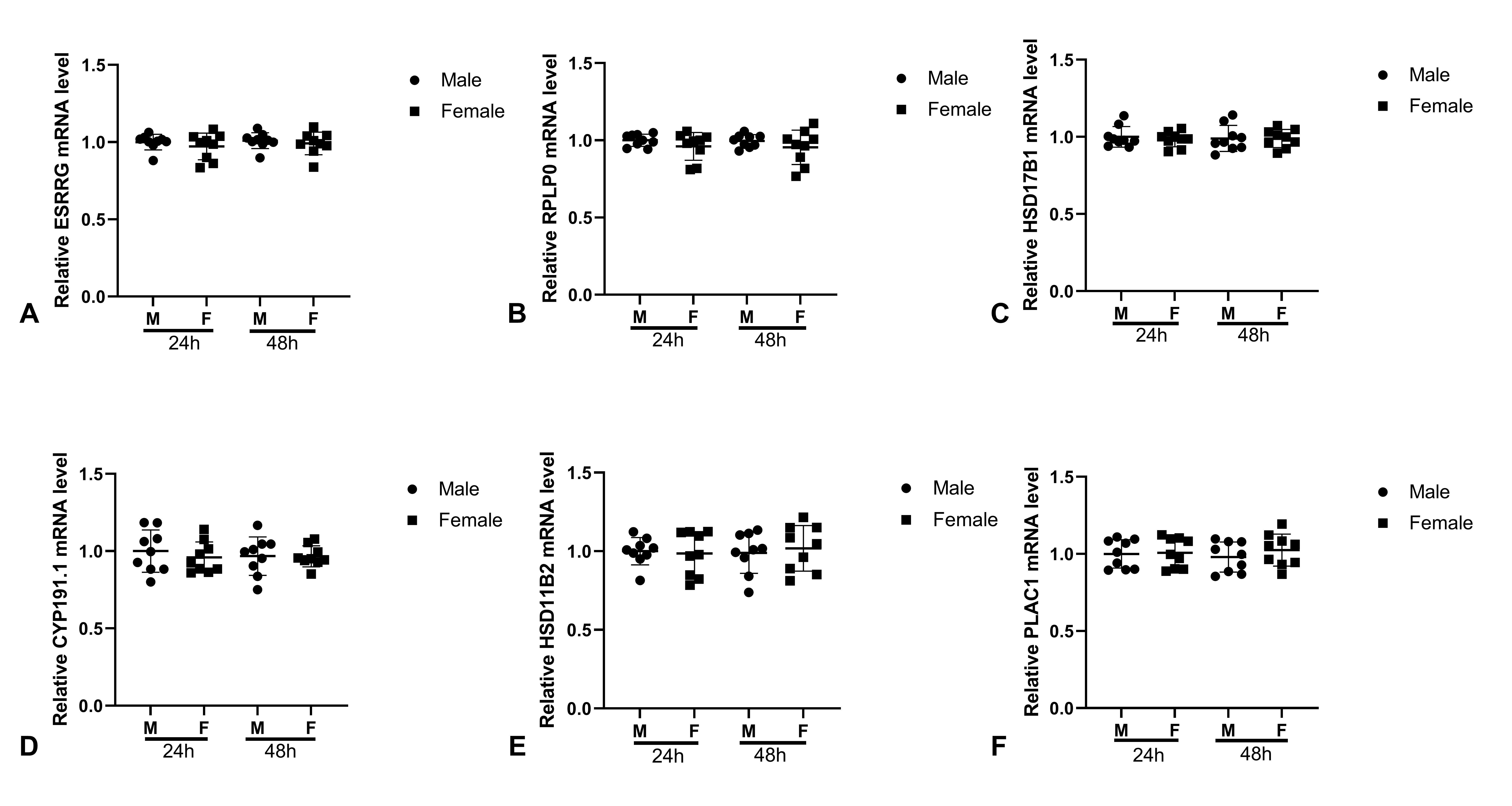

Supplement: ZOU_Sup_2_ioac044 [file zou_sup_2_ioac044.jpeg]

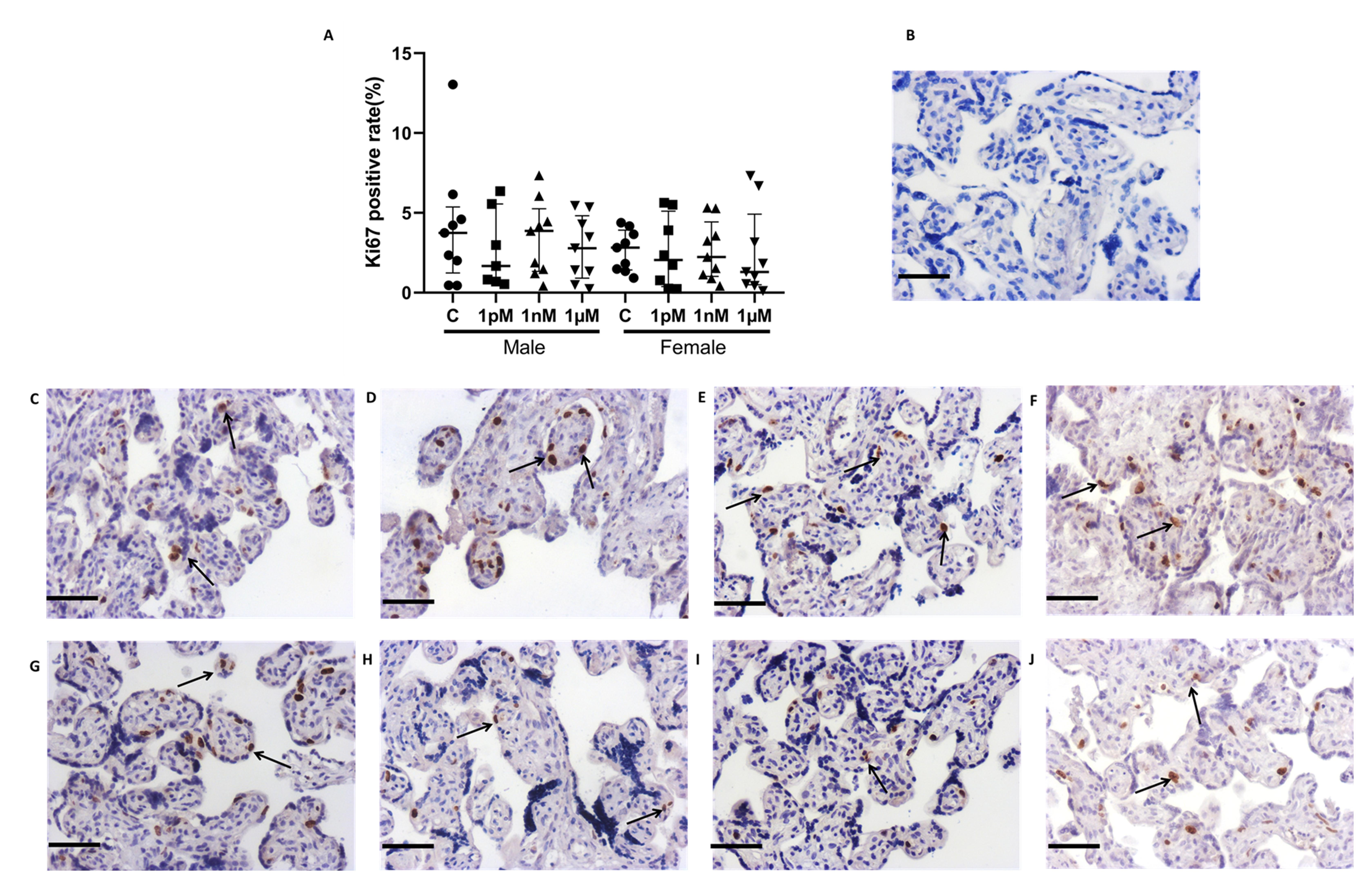

Supplement: ZOU_Sup_3_ioac044 [file zou_sup_3_ioac044.jpeg]

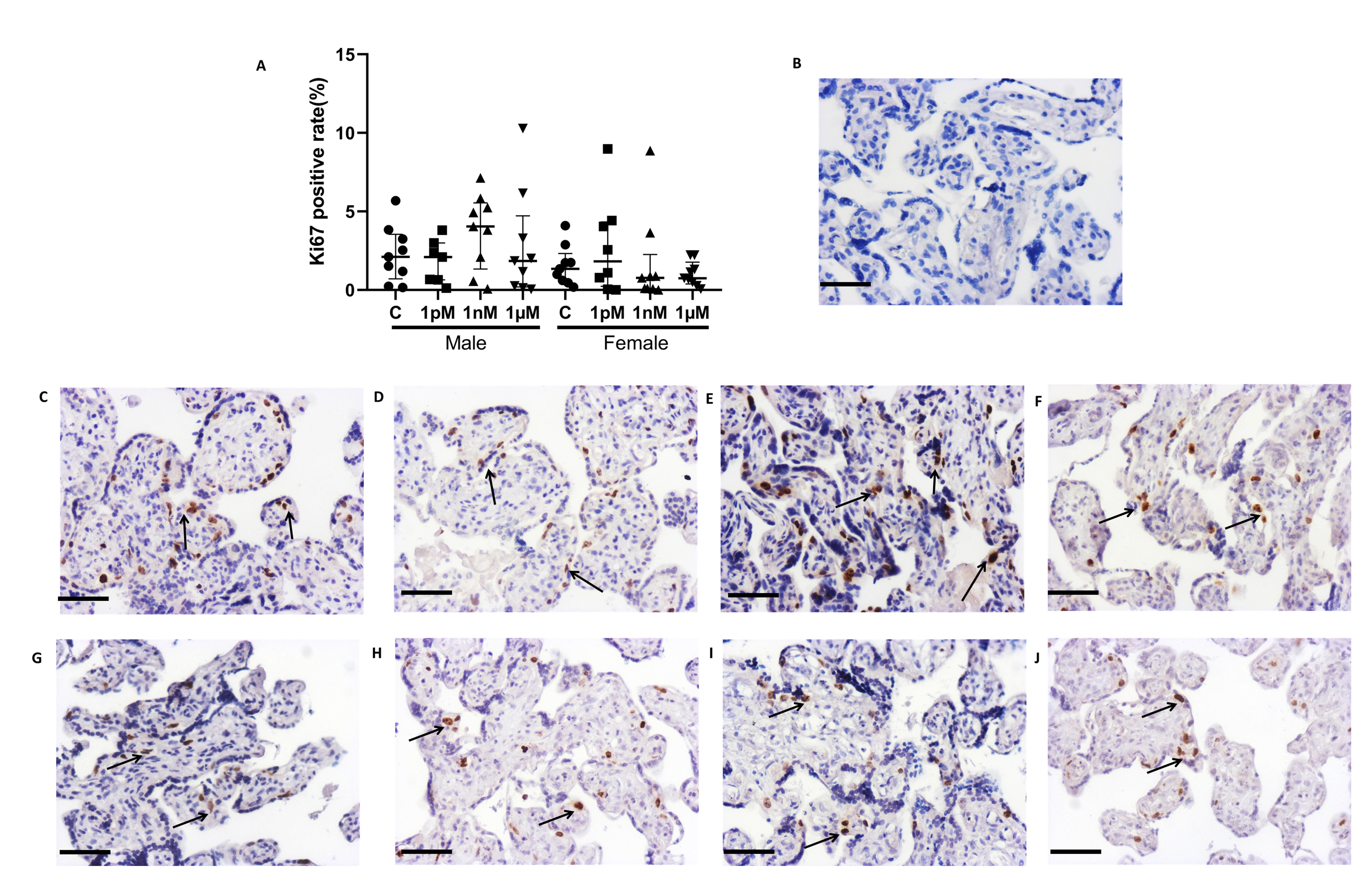

Supplement: ZOU_Sup_4_ioac044 [file zou_sup_4_ioac044.jpeg]

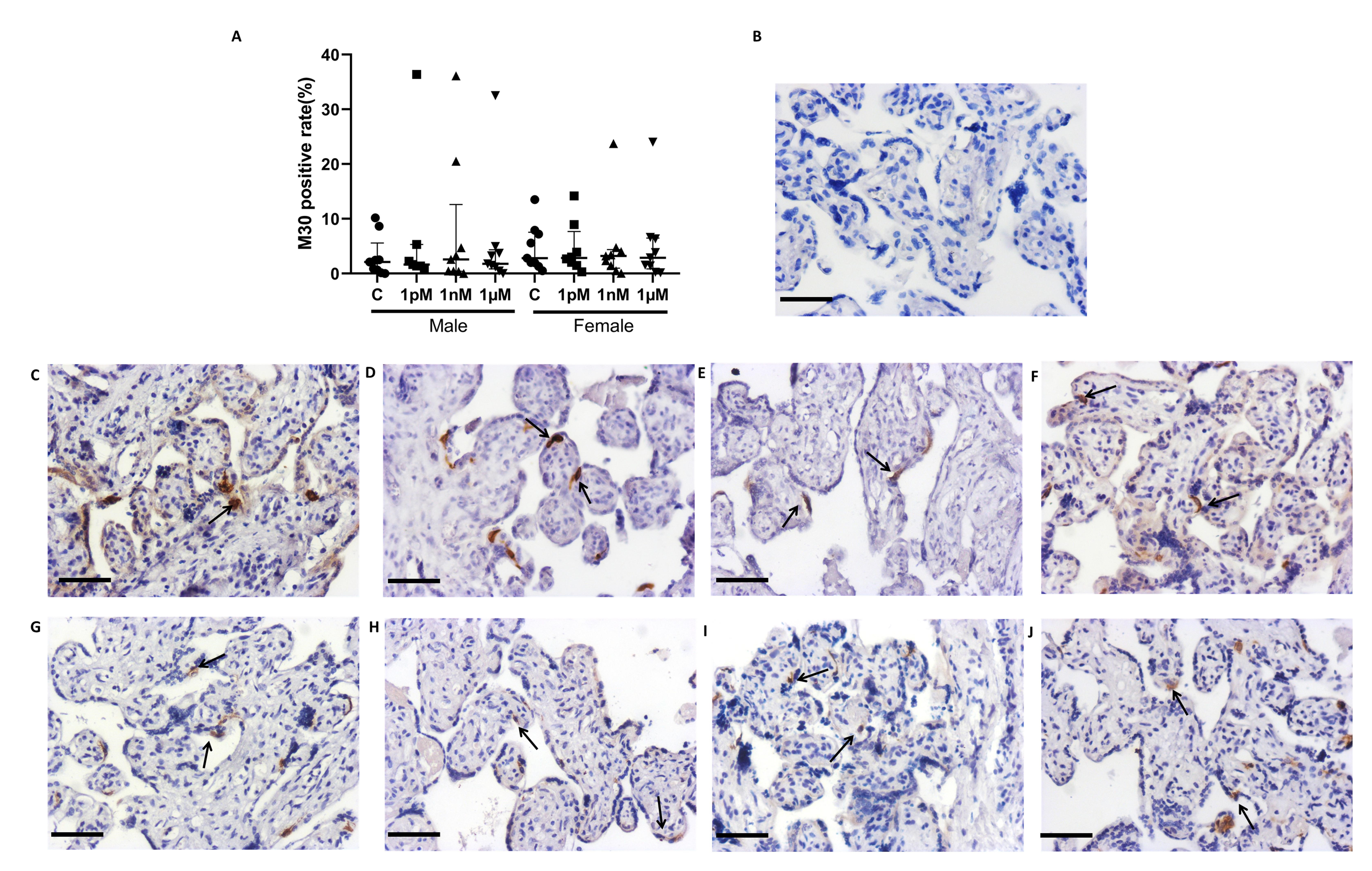

Supplement: ZOU_Sup_5_ioac044 [file zou_sup_5_ioac044.jpeg]

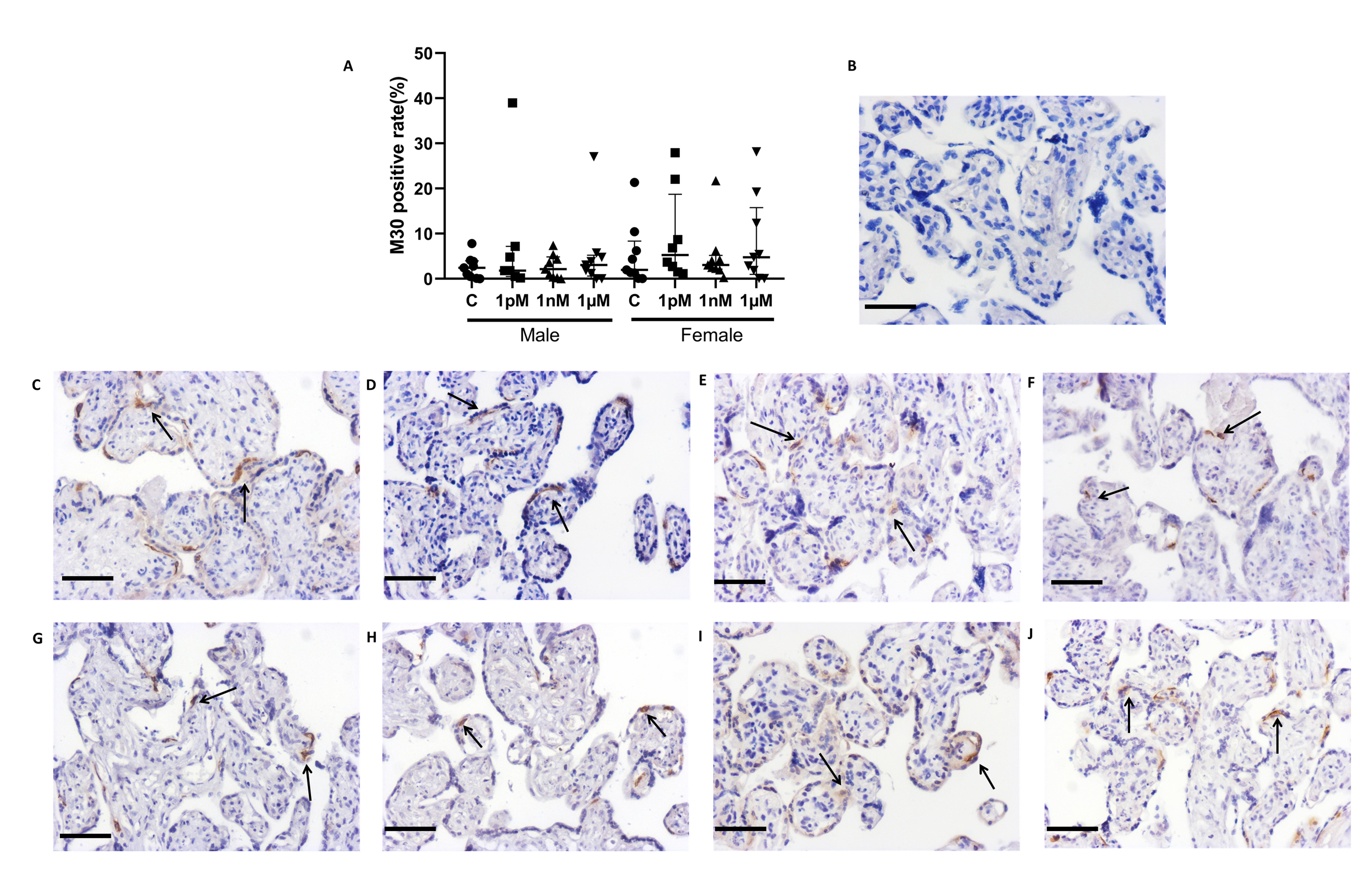

Supplement: ZOU_Sup_6_ioac044 [file zou_sup_6_ioac044.jpeg]

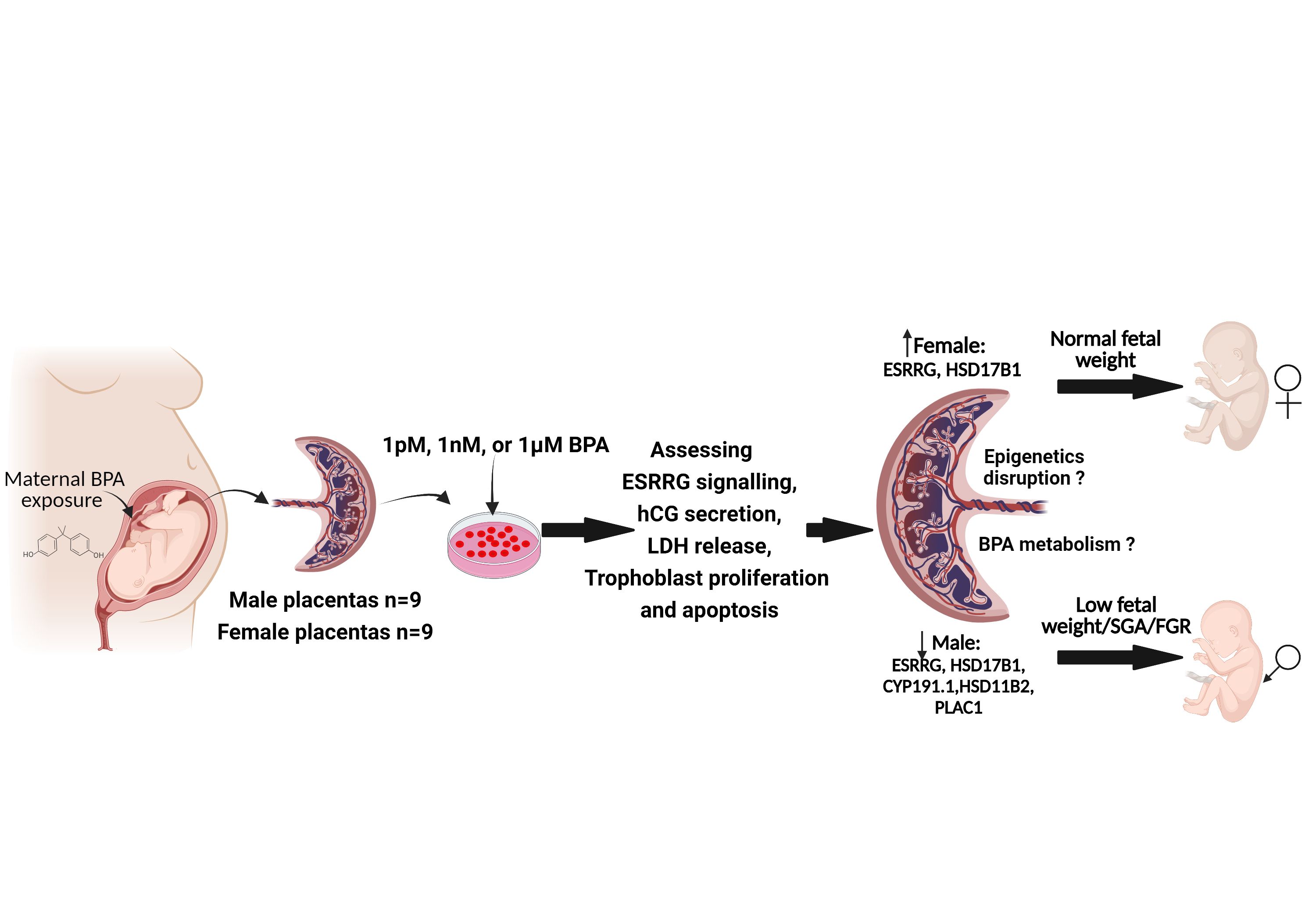

Supplement: ZOU_Sup_7_ioac044 [file zou_sup_7_ioac044.jpeg]
