# Supplementary material for: Sex-specific effects of bisphenol A on the signaling pathway of ESRRG in the human placenta
Source: Biol Reprod. 2022 Feb 26;106(6):1278–91. doi: 10.1093/biolre/ioac044 (PMC9198953; doi:10.1093/biolre/ioac044)
Supplement: supplementary_table_1_ioac044 [file supplementary_table_1_ioac044.docx]

| Primer set | Sequence |
| --- | --- |
| *ESRRG* | Forward: 5’-CTG ACG GAC AGC GTC AAC C-3’  Reverse: 5’-GGC GAG TCA AGT CCG TTC TG-3’ |
| *RPLP0* | Forward: 5’-TGC ATC AGT ACC CCA TTC TAT CA-3’  Reverse: 5’-AAG GTG TAA TCC GTC TCC ACA GA-3’ |
| *HSD17B1* | Forward: 5’-GCC TTC ATG GAG AAG GTG TT-3’  Reverse: 5’-CGA AAG ACT TGC TTG CTG TG-3’ |
| *CYP191.1* | Forward: 5’-ACG GAA GGT CCT GTG CTC G-3’  Reverse: 5’-GTA TCG GGT TCA GCA TTT CCA-3’ |
| *PLAC1* | Forward: 5’-ATT GGC TGC AGG GAT GAA AG-3’  Reverse: 5’-TGC ACT GTG ACC ATG AAC CA-3’ |
| *HSD11B2* | Forward: 5’-GAC CTG ACC AAA CCA GGA GA-3’  Reverse: 5’-GCC AAA GAA ATT CAC CTC CA-3 |
